# Examining social anxiety and dual aspects of social comparison orientation: the moderating role of self-evaluation of social skills

**DOI:** 10.3389/fpsyg.2023.1270143

**Published:** 2023-12-08

**Authors:** Hirohito Okano, Michio Nomura

**Affiliations:** Graduate School of Education, Kyoto University, Yoshida-Honmachi, Japan

**Keywords:** self-evaluation of social skills, social anxiety, social comparison, social comparison orientation, social rank theory of social anxiety, social skills

## Abstract

**Introduction:**

Social comparison orientation comprises ability comparison, which entails superior and inferior ratings; and opinion comparison, which does not include such ratings. Previous research on negative emotions and the social rank theory of social anxiety indicates that social anxiety is positively associated with ability comparison. This is particularly true of individuals with a stronger sense of inferiority (e.g., lower self-evaluation of their social skills). Nevertheless, the relationship between the two aspects of social comparison orientation and social anxiety remains unclear.

**Methods:**

Two hundred thirty-eight individuals (*M*_*age*_ = 40.53 ± 9.78 years, 50.4% men) participated in an online cross-sectional survey questionnaire.

**Results:**

Social anxiety was positively correlated with ability comparison but not opinion comparison. The relationship between social anxiety in situations observed by others and ability comparison was stronger for individuals with lower (vs. higher) self-rated social skills.

**Discussion:**

This study showed that the two types of social comparison are differentially related to social anxiety. The findings support the social rank theory of social anxiety, which states that social comparisons involving superior and inferior ratings lead to social anxiety owing to the perception of one’s inferiority. Making such social comparisons can result in heightened social anxiety, particularly for individuals with low self-evaluations of social skills. The results indicate the importance of these social comparisons in the emergence and persistence of social anxiety. Furthermore, the potential of interventions based on mindfulness, compassion, social media, and video feedback in mitigating the negative effects of such social comparisons is discussed.

## 1 Introduction

People compare themselves with others in various situations, often resulting in emotional consequences (Festinger, 1954). Social comparison orientation is the tendency to compare oneself with others regarding abilities, ideas, and so on. It has two components: ability comparison and opinion comparison (Gibbons and Buunk, 1999). Ability comparison refers to social comparisons based on ability and is made to ascertain an individual’s superiority or competence. Conversely, opinion comparison involves evaluating opinions and ideas, focusing on the opinions and feelings an individual should adopt. Ability comparison is grounded in a competitive mindset, while this might not be the case for opinion comparison (Ozimek and Bierhoff, 2020; Liu et al., 2021).

Between the two factors, only ability comparison is associated with negative emotions such as depression, envy (Park and Baek, 2018), risk-taking (Liu et al., 2021), low self-esteem (Ozimek and Bierhoff, 2020), and social maladjustment (Miao et al., 2018). The two factors of social comparison orientation could exhibit different and unique relationships with other variables and need to be examined separately (Gerson et al., 2017). However, scant research has examined the relationship between social comparison orientation and social anxiety—anxiety that arises in of social or performance situations that may attract the attention of others. A few studies reveal that social anxiety positively correlates with overall social comparison orientation (Gregory and Peters, 2017; Jiang and Ngien, 2020). It is possible to argue that those with high social anxiety have an uncertain self-concept (e.g., Stopa et al., 2010); therefore, they make more social comparisons to clarify their self-concept (Gibbons and Buunk, 1999). However, it is unclear how the dual aspects of social comparison orientation (not the overall score of the scale) are associated with social anxiety. Examining how the two aspects of social comparison orientation relate to social anxiety is essential for a more detailed understanding of the association between general social comparison orientation and high social anxiety shown in previous studies. Thus, we focused on the two aspects of social comparison orientation and hypothesized that only ability comparison is associated with higher social anxiety.

The theoretical studies on social anxiety support our hypothesis that social anxiety positively correlates with ability comparison. According to social rank theory (Trower and Gilbert, 1989), those with high social anxiety view others as competitors for resources rather than as friendly collaborators, which is similar to the mindset behind ability comparison. These individuals compare their social ranks and abilities with those of others (i.e., a form of social comparison). These comparisons result in social anxiety when one’s social rank is low and perceived as inferior. Previous research supports this model (Hope et al., 1998; Haker et al., 2014; Tone et al., 2019; Parsons et al., 2021), showing that feeling inferior owing to social comparison is associated with social anxiety (Mitchell and Schmidt, 2014; Goodman et al., 2021). This type of social comparison involves an assessment of superiority or inferiority (Allan and Gilbert, 1995). Based on these findings and theories, social anxiety is expected to be positively correlated with ability comparison. In contrast to ability comparison, in opinion comparison, the targets of comparison are considered informants or role models, not competitors (Park and Baek, 2018; Yang et al., 2018). Because this is not how socially anxious individuals perceive the world as assumed in social rank theory, we hypothesized that opinion comparison and social anxiety might be negatively or non-correlated.

Furthermore, if social anxiety is positively associated with ability comparison, it is conceivable that the association could be moderated by some type of ability. As an ability to moderate the association between social anxiety and ability comparison, we focused on *social skills* and explored its moderating effect. Social skills refer to the skills needed to achieve social goals in various contexts (Mueser and Bellack, 1998). A low self-evaluation of social skills is linked to high anxiety in social situations, including performance contexts (Segrin, 1996; She et al., 2023). Individuals with high social anxiety have cognitive distortions that lead them to underestimate their social skills (Norton and Hope, 2001). Moreover, social anxiety is rooted in beliefs of social incapability and inferiority (Turner et al., 2003). Patients with social anxiety disorder tend to make more social comparisons regarding social skills than healthy controls (Antony et al., 2005). Social skills fall under the category of abilities; thus, making ability comparisons in the context of social skills might have a significant impact on social anxiety. Individuals with a low self-evaluation of social skills and frequent ability comparisons could perceive their social skills as markedly inferior, resulting in a prominent increase in social anxiety. Thus, the relationship between ability comparison and social anxiety could be moderated by self-evaluations of social skills. However, no studies have examined the interaction between self-evaluation of social skills and ability comparison.

In summary, previous research has found that social anxiety positively correlates with overall social comparison orientation. However, it is unclear which of the two components of social comparison orientation is more relevant to social anxiety. The present study hypothesized that social comparison orientation would only be linked to social anxiety for superior and inferior ability comparisons. Furthermore, it was expected that the association between social comparison with superiority and inferiority ratings (i.e., ability comparison) and social anxiety would be stronger for those with lower self-evaluations of social skills. However, it was unclear how self-evaluation of social skills would affect the association between social anxiety and ability comparison. Thus, this study also examined the moderating effect of self-evaluation of social skills on the relationship between ability comparison and social anxiety.

## 2 Methods

### 2.1 Participants

A cross-sectional questionnaire survey was conducted. An online crowdsourcing platform (CrowdWorks^1^) was used to survey 252 people. The procedure for requesting responses to the questionnaire online is the same as in previous studies such as Jiang and Ngien (2020). To participate in the survey, participants were required to be native speakers of Japanese and at least 18 years old. There were no exclusion criteria. Participants provided demographic data (sex, age, and education) on a page created in Qualtrics^2^ and then completed questionnaires, described below. As the survey was conducted online, the platform ensured that participants could not proceed without completing all values, ensuring that none of the 252 participants who completed the survey had missing values. From these 252 participants, we excluded participants with the same IP address, extremely short response times [−2 standard deviation (*SD*)], and those who responded incorrectly to the attention check. The final sample comprised 238 Japanese adults. Regarding sex, 120 were men (50.4%), 117 were women (49.2%), and one refused to disclose this information. The age range was 18–70 years (*M*_*age*_ = 40.53 ± 9.78 years). The participants’ highest level of education was distributed as follows: 4 participants had completed middle school, 52 had completed high school, 32 had completed junior college or technical college, 138 had a university degree, and 10 had a graduate degree. Two participants preferred not to disclose their highest level of education.

Using G*Power 3.1, a *post hoc* power analysis on the incremental coefficient of determination (*R*^2^) of linear multiple regression analysis was conducted by adding an interaction term (sample size: 238, significance level:.05, power:.80). The analysis revealed that the minimum detectable effect size was *f*^2^ = 0.03. Thus, the sample size in the present study was detectable for small to moderate effect sizes and above (Cohen, 1988). The study was approved by the ethical review board of Kyoto University. All participants provided written informed consent before participating.

### 2.2 Measures

#### 2.2.1 Iowa–Netherlands Comparison Orientation Measure (INCOM)

The INCOM is a commonly used scale that measures people’s social comparison orientation. The original version of this scale was developed by Gibbons and Buunk (1999) and has 11 items with two comparison factors: ability comparison (e.g., “I often compare myself with others concerning what I have accomplished in life”) and opinion comparison (e.g., “I often like to talk with others about mutual opinions and experiences”). The Japanese version of the INCOM, developed by Toyama (2002), has 10 items with the same factors as the original version. The internal consistency, retest reliability, and construct validity of the Japanese version of the INCOM have been confirmed (Toyama, 2002). Responses range from (1) *strongly disagree* to (5) *strongly agree* for each item. Since the opinion comparison factor had only three items and a rather low alpha coefficient (α = .67), the mean inter-item correlation was calculated to confirm the reliability. The value was .41, which is considered good (Clark and Watson, 1995).

#### 2.2.2 Social Phobia Scale (SPS)

The SPS and Social Interaction Anxiety Scale (SIAS; introduced in the next subsection) are commonly used scales measuring different aspects of social anxiety and are often used simultaneously. The SPS assesses the fear of being observed by others (e.g., “I feel awkward and tense if I know people are watching me”). Mattick and Clarke (1998) originally developed the scale with 20 items. The Japanese version was created by Kanai et al. (2004). The internal consistency and criterion-related validity of the Japanese version have been confirmed. Responses range from (0) *not at all true of me* to (4) *extremely true of me* for each item.

#### 2.2.3 Social Interaction Anxiety Scale (SIAS)

The SIAS evaluates fears of interacting in dyads and groups (e.g., “I am tense mixing in a group”). As with the SPS, the original version of this scale was developed by Mattick and Clarke (1998) and translated into Japanese by Kanai et al. (2004), who confirmed its internal consistency and criterion-related validity. The scale has 20 items and responses range from (0) *not at all true of me* to (4) *extremely true of me* for each item.

#### 2.2.4 Social Skills Inventory (SSI)

The SSI is a commonly used scale that measures people’s self-evaluation of their social skills. The original version of this scale was developed by Riggio (1986). The Japanese version of the SSI was developed by Kayano (1988), who confirmed its internal consistency and construct validity. Based on previous research (Strahan, 2003), the Social Control Skills subscale (15 items; e.g., “I can fit in with all types of people, young and old, rich and poor”), which assesses the skills to play appropriate roles and present oneself effectively in diverse social situations (Riggio, 1986), was chosen owing to its association with significantly lower self-evaluations among individuals with high social anxiety. Additionally, this subscale seems to be prone to eliciting feelings of superiority or inferiority when compared to others. Responses range from (1) *not at all like me* to (5) *exactly like me* for each item.

### 2.3 Data analysis

Data analyses were conducted using the R software (v. 4.2.0.). The tests’ statistical significance level (α) was set at .05.

First, descriptive statistics were calculated, followed by a correlation analysis. For testing correlation coefficients, variables are assumed to follow a normal distribution. In cases in which the variables did not meet this assumption, we calculated 95% confidence intervals (CIs) for the correlation coefficients using percentile bootstrap with 10,000 resampling iterations (Pek et al., 2018). This approach allows for a robust interval estimation of correlation and regression coefficients even when the normality assumption is not met. Significance was determined if the CI did not include zero.

Subsequently, we performed moderated multiple regression analyses to identify the moderating effects of self-evaluation of social skills on the relationship between ability comparison and social anxiety (the SPS and the SIAS). If the interaction term in the multiple regression model is significant, simple slope analyses were conducted to examine the relationship between ability comparison and social anxiety among individuals with high (+ 1 *SD*) and low (−1 *SD*) self-evaluations of social skills (a moderating variable). In addition, although not the primary focus of this study, the moderating effects of self-evaluation of social skills on the relationship between opinion comparison and social anxiety were also examined in an exploratory manner. It is assumed that the residuals are normally distributed in regression analysis. Therefore, if the assumption was not met, the 95% CIs of the regression coefficient were determined by percentile bootstrap with 10,000 resampling iterations; they were considered significant if the CIs did not contain zero (Pek et al., 2018).

## 3 Results

Table 1 shows the descriptive statistics (mean, *SD*, skewness, kurtosis, and Cronbach’s alpha) and correlation matrix. Most scales were approximately normally distributed, but the SPS was positively skewed. Therefore, the CIs of the correlation coefficients for the SPS were obtained by the percentile bootstrap method.

**TABLE 1:** Descriptive statistics, internal consistency (Cronbach’s alphas), and correlations among variables.

Variable	*M*	*SD*	*Sk*	*Ku*	α	1	2	3	4	5
1. INCOM	3.10	0.67	0.00	−0.29	.84					
2. Ability	3.03	0.78	0.02	−0.62	.87	.94***				
						[.92, .95]				
3. Opinion	3.26	0.80	−0.21	−0.61	.67	.63***	.33***			
						[.55, .70]	[.21, .44]			
4. SPS	1.07	0.77	0.94	0.66	.94	.34***	.42***	−.04		
						[.21, .46]	[.31, .54]	[−.16, .10]		
5. SIAS	1.84	0.85	0.21	−0.55	.95	.24***	.37***	−.18**	.75***	
						[.11, .35]	[.25, .47]	[−.30, −.06]	[.66, .84]	
6. SSI	2.73	0.65	0.39	−0.08	.88	−.09	−.22***	.26***	−.55***	−.81***
						[−.21, .04]	[−.34, −.10]	[.14, .37]	[−.67, −.44]	[−.85, −.76]

Sk, Skewness; Ku, Kurtosis; INCOM, Iowa–Netherlands Comparison Orientation Measure; Ability, ability comparison factor of the INCOM; Opinion = opinion comparison factor of the INCOM; SPS, Social Phobia Scale; SIAS, Social Interaction Anxiety Scale; SSI, Social Skills Inventory (Social Control subscale). Values in square brackets indicate the 95% confidence interval for each correlation. As the SPS was not normally distributed, confidence intervals for correlation coefficients for the SPS were calculated by the percentile bootstrap method with 10,000 resampling iterations. **p < 0.01, ***p < 0.001.

A correlational analysis was conducted to examine the relationship between the two factors of social comparison orientation and social anxiety. INCOM total scores showed a weak positive correlation with the SPS and the SIAS. Ability comparison showed a moderate positive correlation with the SPS and the SIAS. Contrastingly, opinion comparison did not show a positive correlation with the SPS and the SIAS.

**FIGURE 1:**
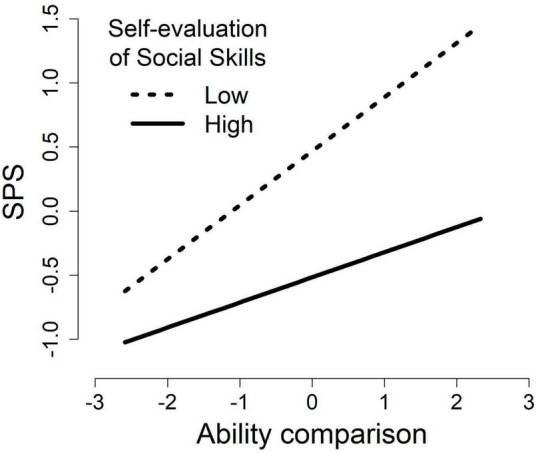
Moderating effect of self-evaluation of social skills on the relationship between ability comparison and the Social Phobia Scale (SPS). SPS, Social Phobia Scale.

Moderated multiple regression analyses were performed to examine whether self-evaluation of social skills moderates the relationship between social anxiety and ability comparison (Table 2). As the moderated multiple regression model for the SPS did not appear to have normally distributed residuals, a Kolmogorov–Smirnov test was performed and it was found that the residuals were not normally distributed (*D* = 0.09, *p* = .04). Therefore, 95% CIs for the standardized regression coefficients were obtained by the percentile bootstrap method, which showed that the interaction was significant (β = −0.11, 95% CI [−0.19, −0.03]). The regression coefficient was significant even when classical tests, rather than bootstrapping, were performed (β = −0.11, *p* = .01, 95% CI [−0.20, −0.03]). Subsequently, a simple slope analysis was conducted to examine the relationship between ability comparison and the SPS among individuals with high (+ 1 *SD*) and low (−1 *SD*) self-evaluations of social skills. Ability comparison and the SPS were positively correlated for low (β = 0.42, 95% CI [0.29, 0.54]) and high (β = 0.20, 95% CI [0.09, 0.30]) self-evaluations of social skills, with a stronger association observed at lower levels of self-evaluation of social skills (Figure 1). Conversely, the moderated multiple regression model for the SIAS did not show a significant interaction (β = −0.03, *p* = .36, 95% CI [−0.09, 0.03]).

**TABLE 2:** Testing moderating effect of self-evaluation of social skills on the relationship between ability comparison and social anxiety (the SPS and the SIAS).

*IV: SPS*					*IV: SIAS*				
Predictor	β	95%CI		*p*	Predictor	β	95%CI		*p*
		*LL*	*UL*				*LL*	*UL*	
(Intercept)	−0.025	−0.126	0.076	.625	(Intercept)	−0.007	−0.079	0.066	.860
Ability	0.309	0.217	0.396	.000	Ability	0.196	0.122	0.269	.000
SSI	−0.493	−0.589	−0.393	.000	SSI	−0.766	−0.840	−0.692	.000
Ability × SSI	−0.113	−0.187	−0.029	.011	Ability × SSI	−0.030	−0.093	0.034	.359

SPS, Social Phobia Scale; SIAS, Social Interaction Anxiety Scale; Ability, ability comparison factor of the INCOM; SSI, Social Skills Inventory (Social Control subscale). Confidence intervals for standardized regression coefficients for the SPS were calculated by the percentile bootstrap method with 10,000 resampling iterations.

Table 3 shows the exploratory results of moderated multiple regression analyses to examine whether self-evaluation of social skills moderates the relationship between social anxiety and opinion comparison. The moderated multiple regression model for the SPS showed significant interaction (β = −0.13, *p* = .01, 95% CI [−0.23, −0.03]). Opinion comparison and the SPS were weakly positively correlated for low (β = 0.23, *p* = .002, 95% CI [0.09, 0.36]) but not for high (β = −0.03, *p* = .73, 95% CI [−0.19, 0.13]) self-evaluations of social skills. Conversely, the moderated multiple regression model for the SIAS did not show a significant interaction (β = 0.02, *p* = .70, 95% CI [−0.06, 0.09]).

**TABLE 3:** Testing moderating effect of self-evaluation of social skills on the relationship between opinion comparison and social anxiety (the SPS and the SIAS).

*IV: SPS*					*IV: SIAS*				
Predictor	β	95%CI		*p*	Predictor	β	95%CI		*p*
		*LL*	*UL*				*LL*	*UL*	
(Intercept)	0.032	−0.076	0.140	.554	(Intercept)	−0.004	−0.082	0.074	.925
Opinion	0.099	−0.011	0.208	.078	Opinion	0.029	−0.050	0.108	.475
SSI	−0.560	−0.670	−0.450	.000	SSI	−0.816	−0.896	−0.737	.000
Opinion × SSI	−0.126	−0.228	−0.025	.014	Opinion × SSI	0.015	−0.059	0.088	.695

SPS, Social Phobia Scale; SIAS, Social Interaction Anxiety Scale; Opinion, opinion comparison factor of the INCOM; SSI, Social Skills Inventory (Social Control subscale).

## 4 Discussion

The study showed that of the two social comparison orientations—ability and opinion comparisons—only ability comparison was positively associated with social anxiety. The association between ability comparison and social anxiety was stronger for individuals with lower self-evaluations of social skills and mitigated for those with higher self-evaluations.

First, general social comparison orientation was positively correlated with social anxiety, in line with previous studies (Gregory and Peters, 2017; Jiang and Ngien, 2020). Notably, when considering social comparison orientation by factor, social anxiety demonstrated a positive association exclusively with ability comparison and not with opinion comparison, a distinction not clarified in previous studies. The findings support previous research (Gerson et al., 2017) that discussed the need to consider the two factors of the INCOM separately. Further, this study builds on Park and Baek’s (2018) research, which demonstrated a positive association between ability comparison and negative emotions, such as depression and envy. It extends our understanding regarding the correlation between social anxiety and ability comparison orientation.

The finding that social anxiety was positively correlated only with ability comparison could be difficult to explain solely based on the theory that socially anxious individuals engage in social comparisons to clarify their unclear self-concept (see Gibbons and Buunk, 1999). According to the social rank theory of social anxiety (Trower and Gilbert, 1989), a possible explanation is that compared to their counterparts, those with high social anxiety make more ability comparisons because they view the people around them as competitors. Indeed, competitiveness is linked to ability comparison (Liu et al., 2021): People with high social anxiety tend to view others as competitive (Tone et al., 2019). This view could account for the high ability comparisons among those who are socially anxious. Contrastingly, those who do not view the people around them as competitive and have low ability comparisons could experience less social anxiety because they have fewer occasions to feel inferior compared to their counterparts. Further, opinion comparison was not positively correlated with social anxiety. In opinion comparison, the subject is viewed as an informant or role model (Yang et al., 2018). This friendly attitude differs from the competitive attitude of socially anxious individuals and is not likely associated with social anxiety.

Further, this finding indicates that social anxiety could mediate the link between ability comparison and social maladjustment among university students (Miao et al., 2018). Performance goals, which rely on social comparison, such as ability comparison, are positively associated with anxiety among college students (Liu et al., 2023). When they view others as their competitors and engage in fierce competition, they may also experience mental health problems via, for example, sacrificing sleep (Cao, 2023). Based on these observations, further research is needed on the effects of competitive attitudes, including ability comparison, on people’s anxiety and mental health.

Self-evaluations of social skills were negatively correlated with social anxiety. This is consistent with previous studies (Segrin, 1996; She et al., 2023). Notably, the relationship between ability comparison and social anxiety in situations observed by others (as measured by the SPS) was stronger when the self-evaluation of social skills was lower. This finding aligns with Trower and Gilbert (1989), who suggested that perceived low social rank, including poor social skills (Zuroff et al., 2010), contributes to elevated social anxiety levels through social comparisons accompanied by superior and inferior evaluations. These results are also consistent with Antony et al.’s (2005) findings, indicating that social comparisons are particularly prevalent in relation to social skills among patients with social anxiety disorder compared to a healthy group. Individuals who perceive their social skills as inadequate might experience stronger social anxiety, as frequent comparisons could reinforce their sense of inferiority compared to those around them. Interestingly, these effects observed in the SPS were not evident in the SIAS. This suggests that frequent awareness of inferiority regarding social skills affects social anxiety in situations scrutinized by others, such as those associated with the SPS. However, this effect was not strong in cases in which individuals interact with others, such as those related to the SIAS. In scrutinized situations (e.g., public speaking), people may be particularly more likely to be aware of their own and others’ social rank than in situations related to the SIAS (e.g., chatting). It is also interesting that, although not the primary focus of this study, the results of the moderated regression analysis for opinion comparison showed a weak positive association between the SPS and opinion comparison only for individuals with low self-evaluations of social skills. Further research on the background mechanism is encouraged.

### 4.1 Theoretical implications

The two aspects of social comparison orientation were differentially associated with social anxiety. This indicates that higher social comparison orientation among those with higher social anxiety is particularly strong on aspects related to superiority and inferiority, supporting the social rank theory of social anxiety. Furthermore, as in Gerson et al. (2017), our results also suggest that future research dealing with social comparison orientation should distinguish between the two aspects. The results also suggest that for social anxiety, both the perception of one’s inferiority as a result of social comparisons and the frequency of social comparisons (i.e., social comparison orientation) should be considered (see Goodman et al., 2021). Future research should examine variables that mediate the association between these two dimensions and social anxiety (e.g., self-esteem).

### 4.2 Practical implications

Interventions that reduce the frequency of social comparisons involving superior/inferior ratings could decrease social anxiety. Comparisons involving superiority or inferiority are generally made in a mindless state (Langer et al., 2010); thus, mindfulness-based interventions could be promising. Compassion-based interventions could also be effective in reducing such comparisons, as the mentality that underlies such comparisons, such as viewing others as one’s competitors, is at odds with a mentality of caring for others (Gilbert, 2015).

Social media and social comparison are deeply interrelated and impact people’s mental health. For example, using social media such as Instagram can lead to social anxiety, and this association is entirely mediated by a general social comparison orientation (Jiang and Ngien, 2020). The present study extends their research and suggests that the impact of social media use on social anxiety can be minimized by suppressing social comparisons, especially those involving superior/inferior evaluations.

The results also indicate that interventions to enhance the self-evaluations of social skills could potentially mitigate the link between high ability comparison and increased social anxiety. One such effective intervention is video feedback (Warnock-Parkes et al., 2017), which improves the self-evaluation of social skills for those who underestimate their own abilities in this regard. Interventions such as this can reduce social anxiety related to social comparisons by improving self-evaluations of social skills.

### 4.3 Limitations

This study reveals novel findings on the relationship between social anxiety, the two factors of social comparison orientation, and self-evaluation of social skills. However, some limitations should be noted. The study was based on a sample of Japanese adults, and the results could differ among people of different nationalities, ages, or clinical groups. Although a relatively diverse age range was represented, the sample did not include those aged younger than 18 years or those older than 70 years. Lastly, owing to the limitations of a cross-sectional design, it would be interesting to explore causal relationships between variables in future research.

## 5 Conclusion

This study deepens our understanding of the complex dynamics between social anxiety, the two factors of social comparison orientation, and self-evaluation of social skills. The findings indicate avenues for further investigation and the potential for interventions in addressing social anxiety. Future research exploring diverse populations and causal relationships will be important in advancing this field of study and informing potential therapeutic approaches.

## Data availability statement

The raw data supporting the conclusions of this article will be made available by the authors, without undue reservation.

## Ethics statement

The studies involving humans were approved by the ethical review board of Kyoto University. The studies were conducted in accordance with the local legislation and institutional requirements. The participants provided their written informed consent to participate in this study.

## Author contributions

HO: Conceptualization, Data curation, Formal analysis, Investigation, Methodology, Project administration, Software, Visualization, Writing-original draft, Writing-review and editing. MN: Funding acquisition, Supervision, Writing-review and editing.

## Footnotes

1
https://crowdworks.jp/

2
https://www.qualtrics.com/

## References

[B1] Allan S. Gilbert P. (1995). A social comparison scale: psychometric properties and relationship to psychopathology. *Pers. Individ. Dif.* 19 293–299. 10.1016/0191-8869(95)00086-L

[B2] Antony M. M. Rowa K. Liss A. Swallow S. R. Swinson R. P. (2005). Social comparison processes in social phobia. *Behav. Ther.* 36 65–75. 10.1016/S0005-7894(05)80055-3

[B3] Cao X. (2023). Sleep time and depression symptoms as predictors of cognitive development among adolescents: a cross-lagged study from China. *Psychol. Rep.* 10.1177/00332941231175833 [Epub ahead of print]. 37164938

[B4] Clark L. A. Watson D. (1995). Constructing validity: basic issues in objective scale development. *Psychol. Assess.* 7:309. 10.1037/1040-3590.7.3.309 PMC6754793 30896212

[B5] Cohen J. (1988). *Statistical Power Analysis for the Behavioral Sciences*, 2nd Edn. Hillsdale, NJ: Erlbaum, 10.4324/9780203771587

[B6] Festinger L. (1954). A theory of social comparison processes. *Hum. Relat.* 7 117–140. 10.1177/001872675400700202

[B7] Gerson J. Plagnol A. C. Corr P. J. (2017). Dimensionality of the Iowa-Netherlands comparison orientation measure and its relationship to reinforcement sensitivity theory. *J. Individ. Differ.* 38 256–264. 10.1027/1614-0001/A000242

[B8] Gibbons F. X. Buunk B. P. (1999). Individual differences in social comparison: development of a scale of social comparison orientation. *J. Pers. Soc. Psychol.* 76 129–142. 10.1037//0022-3514.76.1.129 9972558

[B9] Gilbert P. (2015). The evolution and social dynamics of compassion. *Soc. Personal. Psychol. Compass.* 9 239–254. 10.1111/SPC3.12176

[B10] Goodman F. R. Kelso K. C. Wiernik B. M. Kashdan T. B. (2021). Social comparisons and social anxiety in daily life: an experience-sampling approach. *J. Abnorm. Psychol.* 130 468–489. 10.1037/ABN0000671 34472884 PMC8796168

[B11] Gregory B. Peters L. (2017). Unique relationships between self-related constructs, social anxiety, and depression in a non-clinical sample. *Behav. Change.* 34 117–133. 10.1017/BEC.2017.9

[B12] Haker A. Aderka I. M. Marom S. Hermesh H. Gilboa-Schechtman E. (2014). Impression formation and revision in social anxiety disorder. *J. Anxiety Disord.* 28 133–139. 10.1016/J.JANXDIS.2013.05.001 23774009

[B13] Hope D. A. Sigler K. D. Penn D. L. Meier V. (1998). Social anxiety, recall of interpersonal information, and social impact on others. *J. Cogn. Psychother.* 12 303–322. 10.1891/0889-8391.12.4.303 11261958

[B14] Jiang S. Ngien A. (2020). The effects of Instagram use, social comparison, and self-esteem on social anxiety: a survey study in Singapore. *SM* 6:2488. 10.1177/2056305120912488

[B15] Kanai Y. Sasagawa S. Chen J. Suzuki S. Shimada H. Sakano Y. (2004). Development and validation of the Japanese version of Social Phobia Scale and Social Interaction Anxiety Scale 44. *Jpn. J. Psychosom. Med.* 44 841–850. 10.15064/jjpm.44.11_841

[B16] Kayano J. (1988). An integrated approach to social skills research (I): examination of consistency and validity of SSI. *Hum. Sci. Grad. Course. Kansai. Univ.* 31 1–16.

[B17] Langer E. Pirson M. Delizonna L. (2010). The mindlessness of social comparisons. *Psychol. Aesthet. Create. Arts.* 4 68–74. 10.1037/A0017318

[B18] Liu X. Zhang Y. Cao X. Gao W. (2023). Does anxiety consistently affect the achievement goals of college students? A four-wave longitudinal investigation from China. *Curr. Psychol.* 10.1007/s12144-023-05184-x [Epub ahead of print].

[B19] Liu Z. Elliot A. J. Li Y. (2021). Social comparison orientation and trait competitiveness: their interrelation and utility in predicting overall and domain-specific risk-taking. *Pers. Individ. Dif.* 171:110451. 10.1016/J.PAID.2020.110451

[B20] Mattick R. P. Clarke J. C. (1998). Development and validation of measures of social phobia scrutiny fear and social interaction anxiety. *Behav. Res. Ther.* 36 455–470. 10.1016/S0005-7967(97)10031-6 9670605

[B21] Miao H. Li Z. Yang Y. Guo C. (2018). Social comparison orientation and social adaptation among young Chinese adolescents: the mediating role of academic self-concept. *Front. Psychol.* 9:1067. 10.3389/FPSYG.2018.01067 29997555 PMC6030545

[B22] Mitchell M. A. Schmidt N. B. (2014). An experimental manipulation of social comparison in social anxiety. *Cogn. Behav. Ther.* 43 221–229. 10.1080/16506073.2014.914078 24779421

[B23] Mueser K. T. Bellack A. S. (1998). “Social skills and social functioning,” in *Handbook of Social Functioning in Schizophrenia*, eds Mueser K. T. Trarrier N. (Boston, MA: Allyn & Bacon), 79–96.

[B24] Norton P. J. Hope D. A. (2001). Kernels of truth or distorted perceptions: self and observer ratings of social anxiety and performance. *Behav. Ther.* 32 765–786. 10.1016/S0005-7894(01)80020-4

[B25] Ozimek P. Bierhoff H. W. (2020). All my online-friends are better than me – three studies about ability-based comparative social media use, self-esteem, and depressive tendencies. *Behav. Inf. Technol.* 39 1110–1123. 10.1080/0144929X.2019.1642385

[B26] Park S. Y. Baek Y. M. (2018). Two faces of social comparison on Facebook: the interplay between social comparison orientation, emotions, and psychological well-being. *Comput. Hum. Behav.* 79 83–93. 10.1016/J.CHB.2017.10.028

[B27] Parsons C. A. Alden L. E. Biesanz J. C. (2021). Influencing emotion: social anxiety and comparisons on Instagram. *Emotion.* 21 1427–1437. 10.1037/emo0001044 34928691

[B28] Pek J. Wong O. Wong A. C. M. (2018). How to address non-normality: a taxonomy of approaches, reviewed, and illustrated. *Front. Psychol.* 9:2104. 10.3389/FPSYG.2018.02104/BIBTEX PMC6232275 30459683

[B29] Riggio R. E. (1986). Assessment of basic social skills. *J. Pers. Soc. Psychol.* 51 649–660. 10.1037/0022-3514.51.3.649

[B30] Segrin C. (1996). The relationship between social skills deficits and psychosocial problems: a test of a vulnerability model. *Commun. Res.* 23 425–450. 10.1177/009365096023004005

[B31] She R. Kit han Mo P. Li J. Liu X. Jiang H. Chen Y. (2023). The double-edged sword effect of social networking use intensity on problematic social networking use among college students: the role of social skills and social anxiety. *Comput. Hum. Behav.* 140:107555. 10.1016/J.CHB.2022.107555

[B32] Stopa L. Brown M. A. Luke M. A. Hirsch C. R. (2010). Constructing a self: the role of self-structure and self-certainty in social anxiety. *Behav. Res. Ther.* 48 955–965. 10.1016/J.BRAT.2010.05.028 20800751 PMC3778978

[B33] Strahan E. Y. (2003). The effects of social anxiety and social skills on academic performance. *Pers. Individ. Dif.* 34 347–366. 10.1016/S0191-8869(02)00049-1

[B34] Tone E. B. Nahmias E. Bakeman R. Kvaran T. Brosnan S. F. Fani N. (2019). Social anxiety and social behavior: a test of predictions from an evolutionary model. *Clin. Psychol. Sci.* 7 110–126. 10.1177/2167702618794923

[B35] Toyama M. (2002). The relation between social comparison orientation and psychological traits: development of a Japanese version of the Social Comparison Orientation Scale. *Tsukuba Psychol. Res.* 24 237–244.

[B36] Trower P. Gilbert P. (1989). New theoretical conceptions of social anxiety and social phobia. *Clin. Psychol. Rev.* 9 19–35. 10.1016/0272-7358(89)90044-5

[B37] Turner S. M. Johnson M. R. Beidel D. C. Heiser N. A. Lydiard R. B. (2003). The social thoughts and beliefs scale: a new inventory for assessing cognitions in social phobia. *Psychol. Assess.* 15 384–391. 10.1037/1040-3590.15.3.384 14593839

[B38] Warnock-Parkes E. Wild J. Stott R. Grey N. Ehlers A. Clark D. M. (2017). Seeing is believing: using video feedback in cognitive therapy for social anxiety disorder. *Cogn. Behav. Pract.* 24 245–255. 10.1016/J.CBPRA.2016.03.007 29033532 PMC5627505

[B39] Yang C. C. Holden S. M. Carter M. D. K. Webb J. J. (2018). Social media social comparison and identity distress at the college transition: A dual-path model. *J. Adolesc.* 69:7. 10.1016/j.adolescence.2018.09.007 30278321

[B40] Zuroff D. C. Fournier M. A. Patall E. A. Leybman M. J. (2010). Steps toward an evolutionary personality psychology: individual differences in the social rank domain. *Can. Psychol.* 51 58–66. 10.1037/A0018472

